# Inducing Partner Preference in Mice by Chemogenetic Stimulation of CA2 Hippocampal Subfield

**DOI:** 10.3389/fnmol.2020.00061

**Published:** 2020-04-23

**Authors:** Adi Cymerblit-Sabba, Adam S. Smith, Sarah K. Williams Avram, Michelle Stackmann, Austin C. Korgan, Maria C. Tickerhoof, W. Scott Young

**Affiliations:** ^1^Section on Neural Gene Expression, National Institute of Mental Health (NIMH), National Institute of Health, Bethesda, MD, United States; ^2^Neuroscience Program, Department of Pharmacology and Toxicology, School of Pharmacy, University Kansas, Lawrence, KS, United States; ^3^Systems Neuroscience Imaging Resource, National Institute of Mental Health (NIMH), National Institute of Health, Bethesda, MD, United States; ^4^Neurobiology and Behavior Program, Columbia University, New York, NY, United States; ^5^Center for Alzheimer and Dementia Research, The Jackson Laboratory, Bar Harbor, ME, United States

**Keywords:** partner preference, PVN, dCA2, mice, chemogenetic

## Abstract

Social recognition is fundamental for social decision making and the establishment of long-lasting affiliative behaviors in behaviorally complex social groups. It is a critical step in establishing a selective preference for a social partner or group member. C57BL/6J lab mice do not form monogamous relationships, and typically do not show prolonged social preferences for familiar mice. The CA2 hippocampal subfield plays a crucial role in social memory and optogenetic stimulation of inputs to the dorsal CA2 field during a short memory acquisition period can enhance and extend social memories in mice. Here, we show that partner preference in mice can be induced by chemogenetic selective stimulation of the monosynaptic projections from the hypothalamic paraventricular nucleus (PVN) to the CA2 during the cohabitation period. Specifically, male mice spend more time in social contact, grooming and huddling with the partner compared to a novel female. Preference was not induced by prolonging the cohabitation period and allowing more time for social interactions and males to sire pups with the familiar female. These results suggest that PVN-to-CA2 projections are part of an evolutionarily conserved neural circuitry underlying the formation of social preference and may promote behavioral changes with appropriate stimulation.

## Introduction

The evolution of social behaviors and mating strategies is naturally affected by the environment in which a species has to survive. Specifically, pair bonding is a long-lasting preferential association between two sexually mature adults, often described in the monogamous prairie vole, that includes the formation of partner preference, selective aggression toward unfamiliar conspecifics and bi-parental care of the offspring. Thus, selective affiliation toward a familiar conspecific is an inherent aspect of opposite-sex pair bonding and a critical step in the formation of enduring relationships (Young et al., 2011). While mating styles may differ, prosocial behaviors such as selective affiliation toward a familiar peer are observed across monogamous and even promiscuous voles (Lee et al., 2019). This social selectivity has been suggested to underly the establishment of vole social structure.

In the monogamous prairie voles, partner preference formation is indicated by a selective display of pro-social behaviors such as side-by-side contact (“huddling”) and grooming toward a partner rather than other conspecifics. Male partner preference is often assessed in an established test (PPT; Williams et al., 1992) that includes a long term (3 h) presentation of both the familiar and a novel female to the tested male, following 24 h of cohabitation with the familiar female.

Since laboratory mice (typically *Mus musculus*) generally do not display a monogamous mating style or behavioral characteristics of pair bonding, they are not used to model this behavior. Still, mice can recognize potential genetically attractive (dissimilar at the major histocompatibility complex) mate through odor cues (Penn and Potts, 1999), they are highly prosocial animals demonstrating high-order social interactions (Shemesh et al., 2013) and exhibit a rich repertoire of social behaviors. Moreover, they demonstrate helping-like behavior toward a familiar littermate as well as a novel conspecific (Ueno et al., 2019). This is a behavior that could arise from a desire for social interaction (Silberberg et al., 2014). Typically, however, in short, behavioral tests, they prefer to investigate social novelty. Moreover, in a comparative study, mice tested in the common 3-h PPT failed to show preference toward the familiar conspecific unlike the tested prairie voles (Beery et al., 2018). In contrast, it was also reported that no species difference was found in the short (10 min) social preference test, commonly used to test mice (Beery et al., 2018). Given the many conserved features at the anatomical, cellular, and molecular levels within the microtine rodents, it is possible that the neural substrate of partner preference behavior exists and is suppressed, perhaps epigenetically.

Social recognition enables animals to identify and discriminate between conspecifics and to interact based on experience. It is frequently used in varied social behaviors, such as mate choice (Mateo, 2004; Zala et al., 2004), and is required for species living in complex social systems (Ferguson et al., 2002). Social recognition also has been described as an inherent process in the formation of partner preference in prairie voles, governed by a neuronal mechanism that is suggested to be highly conserved across species (Young et al., 2005, 2011; Choleris et al., 2009) and is context dependent. Specifically, pair-bonded male prairie voles show social recognition for females, whereas single males do not (Zheng et al., 2013; Blocker and Ophir, 2015). These results suggest an interaction between mating status and social context when encoding social identity. Furthermore, multiple brain regions such as the lateral septum and medial amygdala were found to be involved in both social recognition and pair-bonding behaviors through the actions of the neurohormone vasopressin (Avp; Young et al., 2011).

We previously established the critical role of the vasopressinergic projections from the paraventricular nucleus of the hypothalamus (PVN) to dorsal hippocampal CA2 area (dCA2) through its vasopressin 1b receptor (Avpr1b) in the formation of social memories (Wersinger et al., 2002; Smith et al., 2016). Furthermore, we were able to dramatically enhance social memories in mice while stimulating this direct neuronal pathway. Taken together with previous data from Avpr1b knock-out mice showing decreased social motivation (Wersinger et al., 2004) and accumulated evidence supporting vasopressin involvement in affiliative behavior across species (Caldwell et al., 2008; Williams Avram and Cymerblit-Sabba, 2017), we aimed to investigate the effect of stimulating the PVN-to-dCA2 projection on opposite-sex partner preference. We hypothesized that stimulation of the pathway underlying social memory while the mouse is experiencing social reinforcement—specifically, cohabitating and assumed mating with a female conspecific—would result in partner preference.

We used a chemogenetic approach, with the delivery of an excitatory coupled synthetic designer receptor exclusively activated by designer drugs (Gq-DREADDs) *via* a herpes simplex virus (HSV) vector into the dCA2 followed by micro delivery of the designer drug clozapine-N-oxide (CNO, an agonist of the DREADD) through a cannula directly into the PVN before the cohabitation. This allowed a transient activation of the neuronal projections from the PVN to dCA2 at the time of cohabitation. For comparison, since studies in prairie voles suggest that the quality and quantity of the social interactions between a pair contribute to the possibility of partner preference formation (Young, 2003), we also examined the effect of a longer, 6-week period of cohabitation of paired mice (without CNO) followed by co-parenting of the offspring, on partner preference. The DREADD activation resulted in the appearance of partner preference whereas the 6-week cohabitation did not. Our results suggest mice could be used to model and study the complex social behavior of partner preference and may provide an important addition to current approaches.

## Materials and Methods

### Mouse Housing Conditions

All housing and procedures were approved by the Animal Care and Use Committee of the National Institute of Mental Health. Male and female C57Bl/6J mice (6–8 weeks old) were purchased from Jackson Laboratory (Bar Harbor, ME, USA). They were housed in an AAALAC-accredited, specific pathogen-free, vivarium at a constant temperature (~21°C) and humidity (50%) in plastic micro-isolator cages (12” × 6.5” × 5.5”) containing wood chip bedding and cotton nestlets. They were maintained on a 12-h light cycle (lights off at 15:00 h) with *ad libitum* access to food and water. Cages were changed on a bi-weekly basis primarily by the same animal caretaker. All animals used in behavioral experiments were adults that were group-housed with littermates until they were cohabitated (see below).

### Surgical Procedures

#### Viral Delivery and Cannulation

Male mice (7–9 weeks old) were anesthetized with an intraperitoneal injection of tribromoethanol (Avertin^®^, 20 mg/ml solution in sterile normal saline; 0.2 ml per 10 g of mouse weight) and placed into a stereotaxic apparatus. After leveling the head position using bregma and lambda as reference points, the skull was exposed *via* a small incision and holes were drilled bilaterally to target the hippocampal dCA2 subfield (2.18 mm posterior to Bregma, ±2.56 mm lateral to the midline, 1.96 mm below the brain surface). An HSV vector was used to deliver either the excitatory DREADD, hM3D(Gq) fused to a fluorescent marker (mCherry), or the fluorescent marker alone into dCA2. Ten mice in the experimental group were injected with 1 μl of hsv-hEF1a-hM3D(Gq)-mCherry (PVN^Gq^; 5 × 10^9^ units/ml, MIT viral core, Cambridge, MA, USA). Similarly, ten control mice were injected with 1 μl of or hsv-hEF1a-mCherry (PVN^mCherry^; 5 × 10^9^ units/ml, MIT viral core).

Viruses were delivered *via* a 5 μl syringe (26 g, Hamilton, Reno, NV, USA) at a rate of 200 nl/min with a 33 g small gauge RN needle attachment and a Micro4 microsyringe pump (World Precision Instruments, Sarasota, FL, USA). Following the injection, the needle was left for an additional 5 min before slowly retracting it from the brain. The skin was then closed with a wound clip. Following 2 weeks of recovery, cannulae (0315GA-SPC, 5 mm cut; Plastics One, Roanoke, VA, USA) were implanted bilaterally into the PVN (0.82 mm posterior to Bregma, ±0.29 mm lateral to the midline, 4.3 mm below the brain surface). Dummy implants (c3151dc-SPC; 5.5 mm; PlasticsOne) were inserted and covered with dust caps (3030DCF; PlasticsOne). Following a week of recovery, each mouse was paired for 24 h with an ovariectomized and estrogen-primed female. Behavioral testing began when the animals were 10–12 weeks old. Only male mice observed with well-targeted viral expression were included in behavioral data analysis.

#### Ovariectomy

Female mice (6–8 weeks old) were ovariectomized. Briefly, a small dorsal midline incision was made, the muscle wall spread using forceps, and the ovaries were removed. Following 4 weeks of recovery, females were either paired with a cannulated male mouse (PVN^Gq^ or PVN^mCherry^) for 24 h cohabitation or used as an unfamiliar stimulus in the partner preference test described below.

#### Estrogen Priming

Ovariectomized females were each administered 1 μg of estrogen benzoate in 100 μl sesame oil subcutaneously for three consecutive days between the hours of 10:00 and 12:00 PM before the day of pairing with a male.

#### Clozapine-N-Oxide Administration

The designer drug and DREADD agonist, CNO, was manufactured under contract for the National Institutes of Health as part of the Rapid Access to Investigative Drug Program funded by the National Institute of Neurological Disease and Stroke for DREADD studies and is pharmaceutical grade. CNO was diluted in DMSO, and then in sterile saline and stored in the dark at 4°C between uses. CNO solution was used up to 72 h after dilution. All male mice, previously injected with the virus, were administered 18 ng CNO directly into the PVN in a 60 nl volume at 100 nl/min rate, 30 min before the partner pairing (cohabitation; stimulated: PVN^Gq+CNO^ or control: PVN^mCherry+CNO^).

### Behavioral Paradigms

#### Twenty-four Hour Partner Pairing—Cohabitation

Experimental and control groups (10 mice each initially; eight and nine, respectively, after discarding three for inaccurate injections sites), PVN^Gq+CNO^ and PVN^mCherry+CNO^, respectively, were brought to the testing room 30 min before the beginning of the dark cycle and, administered CNO, and placed in a fresh mouse cage. After this 30 min interval, an ovariectomized estrogen-primed female was added to the cage. The video was collected for 4 h to allow the monitoring of interactions. The following morning the female and the cage were searched for sperm plugs.

#### Six-Weeks Partner-Pairing Cohabitation

Six female mice were weighed and each paired with a male mouse for 6 weeks with routine cage changes. Females were weighed weekly to determine likely gestational day (GD). When females had gained at least 30% of their original body weight, they were declared pregnant. Estimations of GD were made from weight gain. Females were monitored daily for the appearance of pups. On the day of birth, or postpartum day (PPD) 0, litters were weighed, counted, and sexed. The number of pups ranged from 4 to 10, and females and sires were allowed to keep their entire litter. Males remained in the cage with females and pups even after pup weaning at ~21 days. The father was tested in the PPT with it mate and a stranger female 2–5 days after weaning.

#### Partner Preference Test

The PPT chamber consisted of three modified transparent mouse cages (12” × 6.5” × 5.5”) separated by 10” of Plexiglas tubing (radius: 4”; Figure 2A). Each mouse cage was filled with fresh bedding for each test session. Metal grid lids were affixed to the tops of the three chambers to prevent experimental mice from leaving the PPT test. Each stimulus mouse was tethered to a corner of one of the outer chambers allowing for full access by the test animal (adapted from Winslow, 2003). Stimulus mice were habituated to the tethering collars for 15–30 min before being placed in the chamber. The location of the partner was counterbalanced between outer cages. The tested mouse was placed in the center cage and allowed free interaction for the 3-h session. Videos of behavioral tests were recorded from above and coded by an observer blind to the identity of the mice using JWatcher Software^1^ (Blumstein et al., 2010). Behavioral analysis consisted of duration in the chamber, sniffing, allogrooming, and side-by-side contact (huddling). Sniffing behavior was defined as the mouse’s nose touching the body anywhere from the anogenital region, including the base of the tail, to the head, including the nose, mouth, and ears. Twenty-four hours after testing, the tested mice were euthanized for histology.

**Figure 1:**
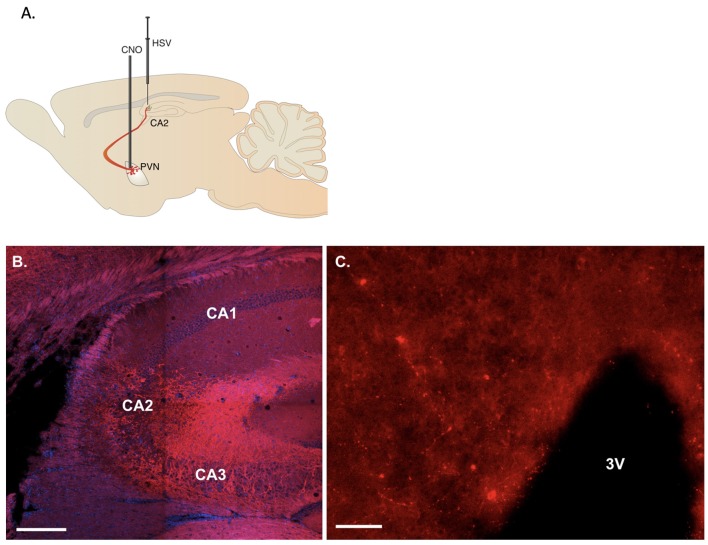
Representative viral labeling. **(A)** Diagram illustrating the viral delivery to dorsal CA2 and subsequent expression in CA2 fibers and paraventricular hypothalamic nucleus (PVN) cells and then their fibers with cannulation for CNO delivery into PVN. **(B)** Coronal section showing the expression of immunoreactive fibers in the hippocampus projecting to dorsal CA2 (scale bar 200 μm). **(C)** Sixteen micrometer coronal section showing sparse cell labeling in the PVN (scale bar 20 μm).

**Figure 2:**
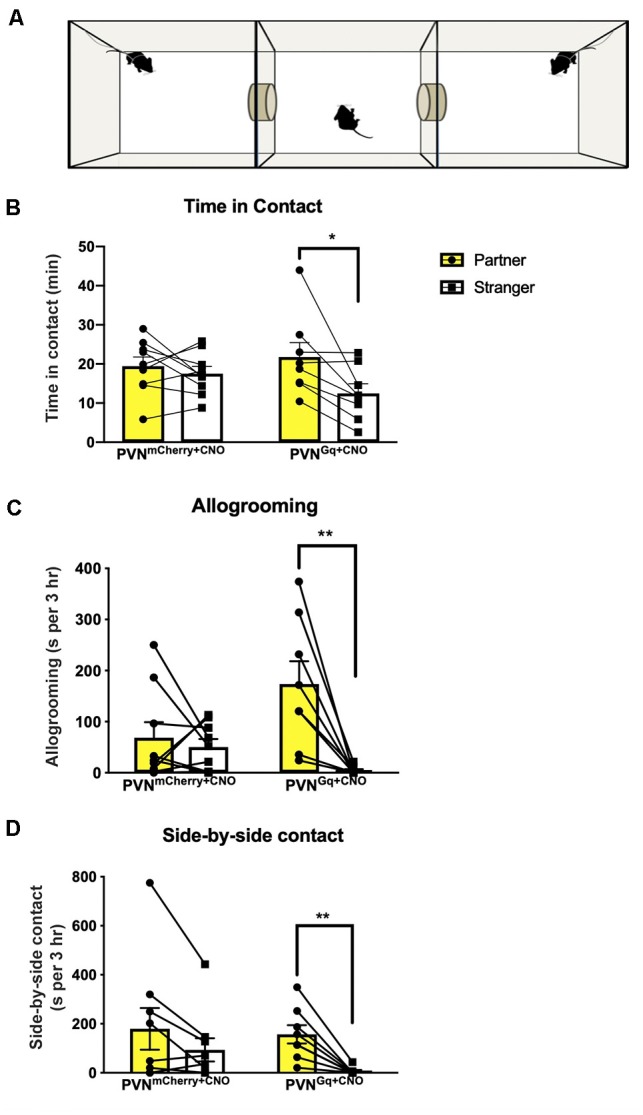
Partner preference induced by CNO treatment. **(A)** Schematic illustration of partner preference test. Tested mice freely explored the chambers while stimulus females were tethered (PVN^Gq+CNO^ mice = 8, PVN^mCherry+CNO^ mice = 9). **(B)** PVN^mCherry+CNO^ mice spent equal time in contact with their partner or the stranger, while PVN^Gq+CNO^ mice demonstrated a significantly longer time in contact with their partner (*P < 0.02). **(C)** PVN^Gq+CNO^ mice spent significantly longer time allogrooming their partners, while the PVN^mCherry+CNO^ group did not (**P < 0.0045). **(D)** PVN^Gq+CNO^ mice spent significantly longer in side-by-side contact (“huddling”) with their partners, while the PVN^mCherry+CNO^ did not (**P < 0.002).

### Histology and Imaging

Animals were anesthetized with isoflurane and transcardially perfused with 4% paraformaldehyde. Brains were removed and post-fixed for 24 h. Following an overnight rinse in 1 M phosphate-buffered saline (PBS), brains were transferred to a 30% sucrose solution for another 24 h after which they were quickly frozen with powdered dry ice. Brains were sliced at 16 um on a cryostat (Leica3050 Biosystems, Buffalo Grove, IL, USA), the sections were mounted onto charged slides, and immunohistochemistry for mCherry amplification was performed. Briefly, sections were incubated at 4° overnight in a rabbit anti-RFP antibody (catalog number 600-401-379, Rockland Antibodies and Assays, Limerick, PA, USA) solution: 10 μl anti-RFP, 800 μl goat serum, 1 ml 20% Triton X-100, and 20 ml PBS. The next day, the sections were rinsed 3 × 10 min in PBS followed by incubation at room temperature for 1 h in the secondary antibody (anti-rabbit, Alexa Fluor 555, Catalog number A27039, Thermo Fisher Scientific, Waltham, MA, USA) solution: 100 μl anti-rabbit, 400 μl goat serum, 1 ml 20% Triton X-100, and 20 ml PBS. The sections were then rinsed in PBS. Sections were imaged using the Zeiss AxioScan Z1 slide scanner and online stitching and shading correction using a 20 × 0.8 NA objective. Images of the hippocampus were collected on a Nikon C2 point-scanning confocal microscope using a 20×, 0.8 NA objective. Z-stacks were collected using a resolution of 1,024 × 1,024 pixels/field and 2× averaging. A maximum projection image is presented.

### Statistical Analyses

Data are presented as mean ± SEM. Behavioral data for CNO treated mice were analyzed by repeated-measures two-way ANOVA with Sidak’s multiple comparisons. The stimulated group PVN^Gq+CNO^ included eight mice and the control group PVN^mCherry+CNO^ included nine mice. For the six mice tested following the 6-week cohabitation, we used Wilcoxon matched-pairs signed-rank test. All statistical tests were carried out using Prism 8 (Graphpad Software Inc.). The level of significance was set to *P* < 0.05.

## Results

### Histology

hM3D(Gq) receptor (tagged with m-Cherry) or m-Cherry were delivered by HSV bilaterally into the dCA2 to allow retrograde transport to projecting neurons, including the PVN, and onset of neuronal expression. We followed viral delivery with cannula implantation to allow selective delivery of the designer drug, CNO, directly to the PVN neurons (Figure 1A). Immunoreactive fibers for mCherry were observed at the site of injection in hippocampal dCA2 (Figure 1B) confirming retrograde transport of the virus to projecting neurons. Although the labeling within the PVN in the 16 μm sections collected was sparse (Figure 1C), labeled cells were observed in multiple sections examined from each mouse.

### Chemogenetic Stimulation of PVN Projections to dCA2 Induces Partner Preference

Following 24 h of cohabitation and verification of sexual contact, mice were tested in a 3-chamber apparatus for partner preference (Figure 2A). Chemogenetic activation of PVN neuronal projections to dCA2 during cohabitation led stimulated mice (PVN^Gq+CNO^) to spend extensively more time in physical contact with their cohabiting partner (mean 21.78 ± 3.6 min) than with the stranger (mean 12.49 ± 2.4 min; Figure 2B). Repeated measures two-way ANOVA of time in contact showed a main effect of stimulus (partner/stranger: *F*_(1,15)_ = 7.778, *P* = 0.0138) and no interaction (group × stimulus: *F*_(1,15)_ *P* = 0.084). *Post hoc* within-group analysis using Sidak’s multiple comparison test showed a significant effect of stimulus (partner/stranger) of *P* < 0.013 in the stimulated group (PVN^Gq+CNO^) and not in the control (PVN^mCherry+CNO^) group (*P* < 0.76).

Stimulated mice (PVN^Gq+CNO^) also spent more time allogrooming their partner (mean 174 ± 44.3 s than the stranger female (mean 4.1 ± 2.8 s; Figure 2C). Repeated measures two-way ANOVA of time spent allogrooming showed a main effect of stimulus (partner/stranger: *F*_(1,15)_ = 12.80, *P* = 0.0028) as well as interaction (group × stimulus: *F*_(1,15)_ = 8.3, *P* = 0.00113) with no main effect of group. *Post hoc* within-group analysis using Sidak’s multiple comparison test showed a significant effect of stimulus (partner/stranger) of *P* < 0.0009 only in the stimulated group (PVN^Gq+CNO^) and not in the control (PVN^mCherry+CNO^) group (*P* < 0.9).

For side-by-side contact, PVN^Gq+CNO^ mice again demonstrated longer sedentary contact with their partner (mean 157.2 ± 37.25 s) than with the stranger (mean 6.2 ± 15.3 s; Figure 2D). Repeated measures two-way ANOVA of side-by-side duration showed a main effect of stimulus (partner/stranger: *F*_(1,15)_ = 19.03, *P* = 0.0006) with no effect of group or interaction (group × stimulus: *F*_(1,15)_ = 1.444, *P* = 0.248). *Post hoc* within-group analysis using Sidak’s multiple comparison test showed a significant effect of stimulus (partner/stranger: *P* < 0.003) only in the PVN^Gq+CNO^ mice but not in the control (PVN^mCherry+CNO^) mice (*P* < 0.07).

**Figure 3:**
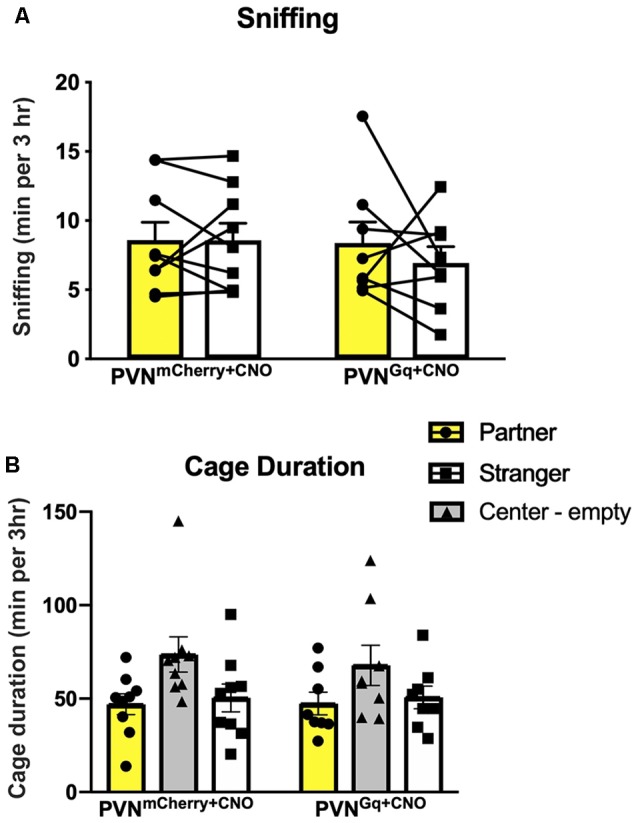
Social behaviors are unchanged by CNO treatment. **(A)** Sniffing duration of either the partner or stranger were similar within and across the tested groups. **(B)** Mice spent similar times in the chambers of the partner or the stranger conspecific in both groups as well as extensive time in the empty center chamber.

There was no difference between groups for sniffing (Figure 3A). Both control and stimulated mice spent similar times sniffing both the partner and the stranger (group × stimulus: *F*_(1,15)_ = 0.5489, *P* = 0.4702). Moreover, stimulated and control mice spent similar amounts of time in the cage of the partner (means of 47.3 ± 6.1 min and 46.9 ± 5.5 min, respectively) as in the cage of the stranger (means of 50.6 ± 6.0 min and 50.41 ± 7.4 min, respectively; Figure 3B). Still, when comparing the time spent in all three cages, including the empty center one, repeated measures two-way ANOVA showed a main effect of a cage (*F*_(1.567,23.51)_ = 3.798, *P* = 0.0465). While the *post hoc* analysis could not identify a significant difference, it seems that both stimulated and control mice spent extensive time in the center empty cage (means of 67.7 ± 10.7 min and 73.6 ± 9.4 min, respectively).

**Figure 4:**
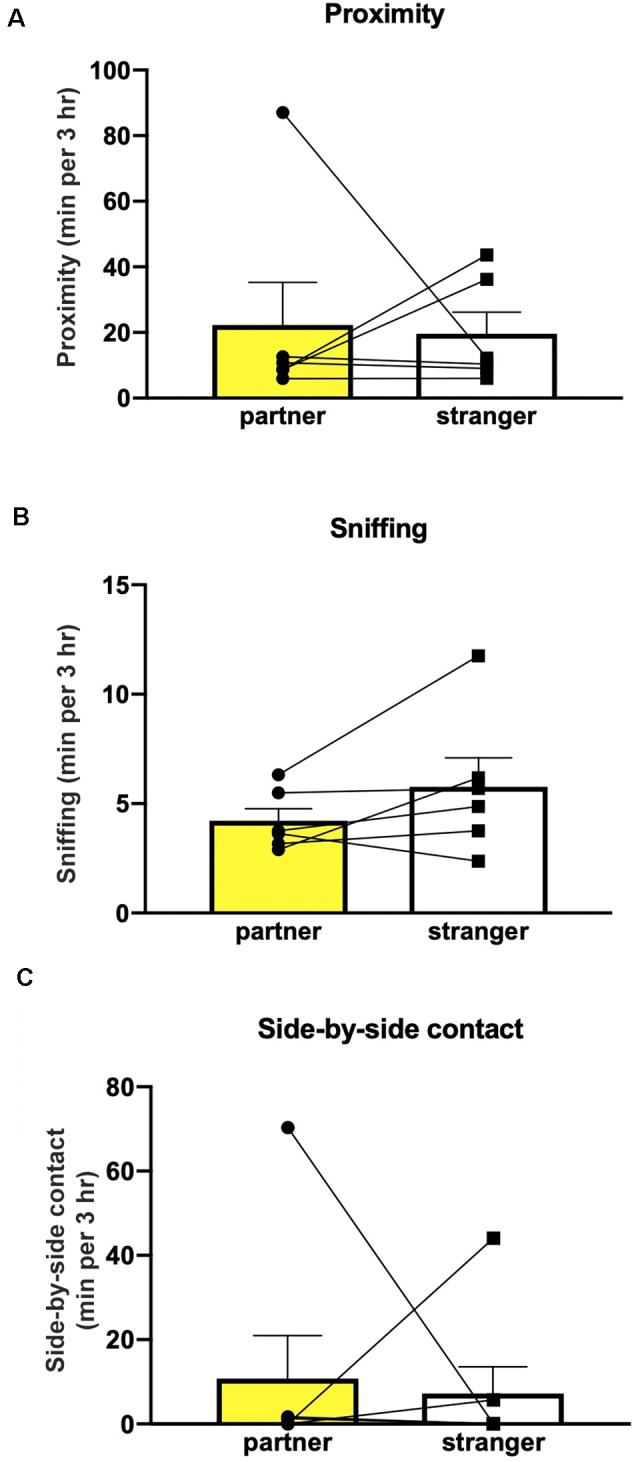
The extended cohabitation period did not induce partner preference. Mice (*n* = 6) did not show preference in sniffing **(A)**, proximity **(B)** or side-by-side contact **(C)**.

Finally, extending the cohabitation period to 6 weeks, in a second experimental group of non-injected mice, as well as allowing the male mice to sire pups to allow a prolonged interaction time, did not affect partner preference in measures of sniffing (Wilcoxon test partner/stranger: *P* = 0.87), proximity (Wilcoxon test partner/stranger: *P* > 0.999) or side-by-side contact (Wilcoxon test partner/stranger: *P* = 0.843; Figure 4).

## Discussion

Studies of neuronal mechanisms underlying partner-directed affiliative behaviors come mostly from prairie voles. Accumulated data suggest these mechanisms involve the integration of social input coming from the conspecific together with reinforcement of the reward system as a result of the interaction. Thus, an interplay between the neuromodulators vasopressin, oxytocin and dopamine is involved. While the neurocircuitry has been intensely investigated, the precise neural mechanisms underlying selective affiliation requires further research (Walum and Young, 2018). All of the behavioral components of pair bonding—selective affiliation toward the partner, biparental care of offspring, and selective aggression defending territory and the partner—require social recognition.

While lab mice do not fully express the complex traits of social monogamy, they are highly prosocial animals demonstrating high-order social interactions (Shemesh et al., 2013) and a rich repertoire of social behaviors. Moreover, although evolutionary processes may lead to different neurochemical profiles in different brain regions (O’Connell and Hofmann, 2012) and epigenetic modifications may occur that are not attributable to changes in DNA sequence (Robinson et al., 2008), the social neural network’s major nodes (McGraw and Young, 2010; Ko, 2017) are similar across the different species. Significant longitudinal changes following social bonding were described in the dorsal hippocampus of prairie voles (López-Gutiérrez et al., 2019). Also, within the social network, the CA2 subfield of the hippocampus is a key player in the modulation of social behaviors (Ko, 2017). Together with evidence of its receptor repertoire that includes Avpr1b, Oxtr (Pagani et al., 2015; Williams Avram and Cymerblit-Sabba, 2017; Piskorowski and Chevaleyre, 2018) and dopamine receptors (Gangarossa et al., 2012), the CA2 is a potential candidate to be part of the neural circuitry underlying partner preference.

Surprisingly, while neuronal labeling appeared to be sparse in the PVN, mice expressing the excitatory Gq and treated with CNO, delivered directly into the PVN during cohabitation, demonstrated increased time in contact as well as allogrooming and side-by-side sedentary contact with their partner, unlike their littermates expressing the mCherry tag alone. These are the cardinal measures of partner preference used in this behavioral paradigm when performed in voles. The enhanced partner preference behavior in the stimulated mice (PVN^Gq+CNO^), appears to be dependent on the coupling of excitatory inputs from the PVN to dCA2 with the 24 h cohabitation with the female. The connections of CA2 with multiple brain regions involved in social and non-social behaviors, such as dentate gyrus, CA1, CA3, septum and median raphe (Cui et al., 2013; Benoy et al., 2018) place it in a position of a network hub. The mechanism by which CA2 interfaces with the different brain systems to facilitate several socially related behaviors requires more research, including how it may be involved in partner preference as our study suggests. Studies to see if the dCA2 is necessary for pair-bonding in prairie voles, for example, would be worthwhile as well.

The lack of partner preference in our 6-week cohabitating and mated mice allows us to conclude that social context manipulation alone is not sufficient to change the typical behavior in the lab mice, emphasizing the need for a direct stimulation of a specific neuronal pathway to produce the partner preference. The lack of monogamy seen in mice is likely a result of evolutionary changes leading to different biological valence of the social context. Phylogenetic studies suggest polygamy in mice maximizes evolutionary fitness by allowing for higher paternity success and stimulating selection competition that results in higher-quality offspring (Emlen and Oring, 1977; Firman and Simmons, 2012). Moreover, they agree with a previous study, comparing partner preference between mice and voles (Beery et al., 2018).

We previously reported that optogenetic stimulation of vasopressinergic fibers in the dCA2 resulted in enhanced social memory, as measured by a difference in sniffing durations. The lack of difference in the sniffing behavior may arise from the limitation of the particular behavioral paradigm. In mice, social memory or approach is typically tested in brief (5–10 min) encounters with the conspecific stimuli, in which mice demonstrate recognition of a familiar conspecific by sniffing it less than the novel conspecific. These differences in duration are typically less than 60 s. In the current study, mice were presented with a familiar partner and a novel female for a prolonged period (3 h). This large increase in the testing time could conceal changes in sniffing. Also, while individual recognition is required behavior for the development of selective affiliation and pair-bonding in voles (Young et al., 2011) when they are tested for social recognition in a social preference test with a short 10-min encounter, voles spend similar times investigating either the familiar or the novel stimulus (Beery et al., 2018). Thus, while the partner preference test allowed us to investigate relevant affiliative behaviors, it may limit the ability to compare recognition memory to the commonly used mouse paradigms. Moreover, the lack of difference in the time spent in each chamber could arise from the mouse’s exploratory behavior. Beery et al. (2018) showed that the mice were more active than the voles in the partner preference test and often crossed into the center chamber. We also observe that the mice tend to spend a greater percentage of time in the center chamber rather than in either of the outer chambers.

## Conclusion

Our results demonstrate that the underlying neurocircuit of partner preference exists in non-monogamous lab mice, and appropriate stimulation may induce this species-atypical behavior. As has been suggested by previous studies, although the social neural network is distributed across multiple brain regions, social interaction can be modulated by manipulating a single key element (Lim et al., 2004; Chen and Hong, 2018). Thus, our study promotes the needed evolutionary and comparative investigations of affiliative behaviors and their underlying mechanisms across and within species exhibiting different social organizations and behaviors.

## Data Availability Statement

The datasets generated for this study are available on request to the corresponding author.

## Ethics Statement

The animal study was reviewed and approved by Animal Care and Use Committee, Division of Intramural Research, National Institute of Mental Health.

## Author Contributions

AC-S: experimental design, surgeries for viral delivery, data analysis, manuscript preparation. AS: experimental design, surgeries for cannula placement, behavioral data collection and analysis. SW: experimental design, behavioral data collection and analysis, image data collection. MS, AK and MT: behavioral data collection and analysis. MT: behavioral data collection and analysis. WY: experimental design and manuscript preparation.

## Conflict of Interest

The authors declare that the research was conducted in the absence of any commercial or financial relationships that could be construed as a potential conflict of interest.

## Footnotes

^1^http://www.jwatcher.ucla.edu/
